# Salicylate Poisoning and Rebound Toxicity

**DOI:** 10.7759/cureus.60241

**Published:** 2024-05-13

**Authors:** Sindhu Harika Peketi, Pratap Kumar Upadrista, Bair Cadet, Johanne Cadet, Noonkee Cadet

**Affiliations:** 1 Internal Medicine, Nassau University Medical Center, East Meadow, USA; 2 Nephrology, Nassau University Medical Center, East Meadow, USA; 3 Nursing, New York City (NYC) Health Plus Hospitals South Brooklyn Health, New York, USA; 4 Nursing, Texas Department Criminal Justice Hospital, Galveston, USA

**Keywords:** aspirin overdose, acetylsalicylic acid overdose, hemodialysis, rebound toxicity, salicylate poisoning

## Abstract

Salicylate exposure and toxicity are associated with a myriad of symptoms and signs, and a comprehensive knowledge of diagnosing and treating salicylate poisoning is needed. Here, we present a case of a 29-year-old female with a past medical history of schizoaffective disorder and bipolar disorder with multiple suicide attempts brought to our hospital, Nassau University Medical Center, East Meadow, by the Emergency Medical Service (EMS) due to an intentional overdose of 300 pills of acetylsalicylic acid. She had mixed acid-base disturbance with respiratory alkalosis and metabolic acidosis. She was started on bicarbonate infusion in the emergency department to maintain a blood pH of 7.5 and to maintain a urine pH of more than 7.5. As her salicylate levels were 98.2 at admission with altered mental status, she was started on slow, low-efficiency hemodialysis. A few hours later, she developed a rebound increase in salicylate levels to 129, associated with a change in mental status and the patient was more confused. She was started on regular hemodialysis with improvement in mental status and elimination of salicylate steadily. Given the extensive nature of toxic effects, a patient with severe salicylate toxicity can deteriorate rapidly and can be challenging to manage. As there is no specific antidote for aspirin, the goals of therapy depend primarily on limiting the absorption of salicylate, enhancing elimination, and providing supportive care. Monitoring the acid-base status and serum salicylate levels closely and monitoring for rebound increase in salicylate levels is of paramount importance. Aggressive hydration to maintain euvolemia, alkalinization, aggressive replenishment of potassium and magnesium, activated charcoal to decrease absorption, and hemodialysis remain the cornerstones of treatment.

## Introduction

Aspirin is an easily available over-the-counter medication. Salicylate, a metabolite of aspirin, is responsible for its toxic effects [1]. Every year, in the United States, around 20,000 salicylate exposures and 50 to 70 fatalities are reported [2]. Given the high rate of exposure and deaths and the myriad of symptoms and signs associated with salicylate toxicity, a comprehensive knowledge of diagnosing and treating salicylate poisoning is needed. Here, we describe a 29-year-old female who presented with an intentional overdose of acetylsalicylic acid and required hemodialysis. Our aim in this report is to present the treatment modalities of salicylate toxicity, the variability in the concentration of plasma salicylate concentrations with time, and the efficacy of therapeutic interventions on it.

## Case presentation

A 29-year-old female with a past medical history of schizoaffective disorder and bipolar disorder with multiple suicide attempts was brought to our hospital, Nassau University Medical Center, East Meadow, by Emergency Medical Service (EMS) due to an intentional overdose of 300 pills of acetylsalicylic acid of strength 325 mg three to four hours before the arrival at our emergency department following, which she developed generalized abdominal pain and nonbilious and non-bloody vomiting. She denied vertigo and tinnitus. On arrival, she was ill-appearing, awake, confused, tachypneic, was dry heaving, had tachycardia at 153 beats per minute, Blood pressure of 146/92 mm hg, afebrile at 99.2, and was saturating at 97% on room air. On evaluation, she had mixed acid-base disturbance with respiratory alkalosis and metabolic acidosis, as mentioned in Table 1.

**Table 1:** Lab values of the patient at admission with reference range Pco_2_: Partial pressure of carbon dioxide, Po_2_: Partial pressure of oxygen, BUN: Blood urea nitrogen, SGOT: Serum glutamic-oxaloacetic transaminase, SGPT: Serum glutamate pyruvate transaminase, INR: International normalized ratio, PTT: Partial thromboplastin time.

Parameter	Value at admission	Normal reference range
pH	7.542	7.35-7.45
Pco_2_	24.5 mm Hg	35.0-45.0 mm Hg
Po_2_	245.0 mm Hg	83.0-108.0 mm Hg
Bicarbonate	15 mmol/L	20 – 31 mmol/L
Anion Gap	16	5-15
Urine Ph	7.6	4.5-8.0
Salicylate	98.2 mg/dL	< 4.0 mg/dL
Sodium	145 meq/L	136-145 meq/L
Potassium	3.6 meq/L	3.5-5.1 meq/L
Magnesium	1.8 mg/dL	1.6-2.6 mg/dL
Phosphorous	5.4 mg/dL	2.4 – 5.1 mg/dL
Lactate	1.8 mmol/L	0.5-1.6 mmol/L
Creatinine	1.1 mg/dL	0.6-1.0mg/dL
BUN	18 mg/dL	9-23 mg/dL
SGOT	31 U/L	7-40 U/L
SGPT	13 U/L	13-40 U/L
Glucose	186 mg/dL	74-106 mg/dL
INR	1.2	0.8-1.1
PTT	40.8	22-36 msec

**Figure 1:**
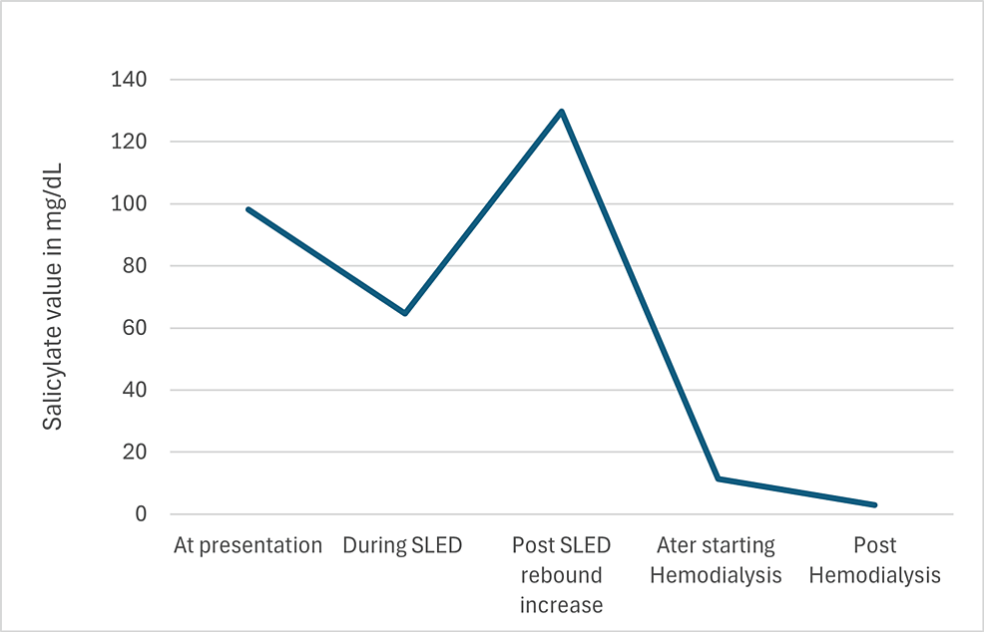
Salicylate levels over time

EKG was suggestive of sinus tachycardia at 129 beats per minute with a prolonged QTc of 501 ms. The chest X-ray was not suggestive of pulmonary disease. As the patient was confused and was vomiting, activated charcoal was not administered as per poison control recommendations. She was started on bicarbonate infusion in the emergency department to maintain a blood pH of 7.5 and to maintain a urine pH of more than 7.5. As her salicylate levels were 98.2 mg/dL at admission with altered mental status, she was started on slow low-efficiency hemodialysis. Post dialysis, her salicylate levels improved gradually to 64.7 mg/dL along with some improvement in acidosis; however, a few hours later, she developed a rebound increase in salicylate levels to 129 mg/dL associated with a change in mental status and the patient was more confused. She was started on regular hemodialysis and later switched to sustained low-efficiency dialysis (SLED), together for a total of eight hours to effectively remove salicylate. Acid-base status, electrolytes, and salicylate levels are monitored every two to four hours. After the dialysis, the patient’s salicylate levels slowly started trending from 129 mg/dL mg down to 55.3 mg/dL and to 11.3 mg/dL and acidosis resolved with bicarbonate improving to 25 meq/L along with improvement in renal function to 0.6 mg/dL, mental status (as shown in Figure 1 and Table 2).

**Table 2:** Depicting timeline of salicylate level, pH, bicarbonate level at presentation and changes with dialysis SLED: Sustained low-efficiency dialysis

Timeline at presentation	Salicylate value in mg/dL	pH	Bicarbonate
At presentation	98.2	7.5	15
During SLED	86.8 -82.3 – 64.7		25 – 24 - 21
Post-SLED rebound increase	129.7	7.7	15
After starting hemodialysis	55.3 – 11.3	7.5	25
Post hemodialysis	< 4.0		29

## Discussion

Assessing the severity of salicylate poisoning should not be solely focused on serum salicylate levels, instead, it should be done in the clinical context of clinical symptoms, signs, and acid-base status. Time lag in serum salicylate concentration due to enteric absorption and differential tissue distribution might cause a jump in the serum salicylate after the initial presentation even without repeated exposure [3-5]. At therapeutic dosing, aspirin is rapidly absorbed from the jejunum with peak blood concentrations achieved in one hour for regular aspirin but delayed up to four and 14 hours with enteric-coated or delayed-release formulation. At therapeutic concentrations, 90% of salicylate is bound to protein and stays in the intravascular compartment, however in overdose patients, protein binding becomes saturated, and the free fraction of salicylate leaves the intravascular compartment and enters vulnerable tissues like the Central Nervous System (CNS).

Salicylate interferes with cellular metabolism and causes metabolic acidosis, hyperthermia, and fluid losses. It activates the respiratory center of the medulla resulting in tachypnea, hyperventilation resulting in respiratory alkalosis, increased renal loss of bicarbonate, and increased insensible fluid loss. Even though the initial presentation is respiratory alkalosis, high anion gap metabolic acidosis due to the accumulation of lactic acids and ketoacids soon develop.

In patients with severe toxicity, cardiorespiratory centers are completely inactivated, and cerebral edema can occur. Other effects include depleting glycogen stores and impairs gluconeogenesis, causes a catabolic state resulting in hypoglycemia, and accumulation of organic acid and ketoacids. Salicylate inhibits cyclooxygenase and contributes to platelet dysfunction and gastric mucosal injury. Tinnitus due to damage of cochlear cells is an early and characteristic symptom that can occur at even therapeutic concentrations. Other characteristic manifestations include fever, vertigo, and blurry vision.

Serum salicylate concentrations range from 30 to 50 mg/dL in mild toxicity, 50 to 90 mg/dL in moderate toxicity, and > 90 mg/dL in severe toxicity [6]. Blood pH is normal or alkalotic in mild cases and alkalotic in moderate toxicity while acidic in severe toxicity with alkaline urine (pH> 5). Given the extensive nature of toxic effects, a patient with severe salicylate toxicity can deteriorate rapidly and can be challenging to manage. As there is no specific antidote for aspirin, the goals of therapy depend primarily on limiting the absorption of salicylate, enhancing elimination, and providing supportive care [7].

Rapidly assessing airway, breathing, and circulation are the primary goals of any poisoning patient. Avoid intubation if possible as salicylate-induced respiratory alkalosis is blocked by paralytics during intubation. This will cause the acidosis to worsen acutely, and it promotes salicylate anions to convert into salicylic acid and redistributing it into tissues and exacerbating toxicity thus contributing to increased peri-procedural cardiovascular collapse and cardiac arrest [8].

Irrespective of the time since ingestion unlike other toxic ingestions, at least one dose of activated charcoal (1g/kg) should be given to prevent further intestinal absorption of the salicylate [9]. However, AC should not be given to patients who have altered mental status and are unable to protect their airways. Intubating for administering AC should be avoided as spontaneous respiration by providing high-minute ventilation provides greater clinical benefit than AC. Our patient had altered mental status that put her at higher risk of aspiration and placing a nasogastric tube or intubating her for the activated charcoal would be more detrimental than beneficial.

Urinary alkalinization to keep urinary pH more than 7.5 is the second most effective method in non-oliguric patients to increase ionized salicylates and enhance salicylate excretion after hemodialysis. Ionized salicylates are more easily excreted by the kidneys and less likely to penetrate and accumulate in tissue than the nonionized form. While urinary alkalinization significantly increases elimination, serum alkalinization decreases detrimental tissue distribution like CNS toxicity [10-13]. Glucose supplementation is needed to avoid CNS hypoglycemia even in serum normoglycemia, as cerebral glycolysis outpaces serum glycolysis. Guidelines for optimal alkalinization suggest an initial dose of sodium bicarbonate (NaHCO3) of 1 to 2 mEq/kg as a bolus followed by continuous infusion of 100 to 150 ml/h of 100 to 150 mEq of NaHCO_3_ mixed in 1L of 5% dextrose. Alkalemia from primary respiratory alkalosis is not a contraindication to give NaHCO_3_ and can be given till an arterial goal of pH of 7.50 to 7.55 and urinary pH of more than 7.5. However, regular monitoring of blood gases and serum salicylate levels every two hours along with hourly urinary pH is needed. Once serum salicylate concentration is less than 40 mg/dL the patient is asymptomatic with normal respiratory rate and effort and acid-base status has normalized, alkalinization therapy can be stopped [10-13].

Apart from acute toxicity with serum salicylate concentration more than 100 mg/dl with normal renal function or a serum concentration of 90 mg/dL with impaired renal function, alteration in mental status, hyperthermia, seizure, marked acidemia despite aggressive resuscitation, cerebral or pulmonary edema, fluid overload limiting administration of bicarbonate infusion, clinical deterioration, AKI and serum salicylate concentrations raising or unchanged despite bicarbonate infusion are indications for hemodialysis [14,15].

## Conclusions

This case highlights the significance of closely monitoring acid-base status and serum salicylate levels and monitoring for rebound increase in salicylate levels. A patient with salicylate toxicity can deteriorate rapidly, so a high level of caution is needed to limit further absorption of salicylate, enhance elimination, and provide supportive care. Aggressive hydration to maintain euvolemia, Alkalinization, aggressive replenishment of potassium and magnesium, activated charcoal to decrease absorption, and hemodialysis remain the cornerstones of treatment.
